# Supplementary figures and images for: Metabotypes of Pseudomonas aeruginosa Correlate with Antibiotic Resistance, Virulence and Clinical Outcome in Cystic Fibrosis Chronic Infections
Source: Metabolites. 2021 Jan 21;11(2):63. doi: 10.3390/metabo11020063 (PMC7909822; doi:10.3390/metabo11020063)

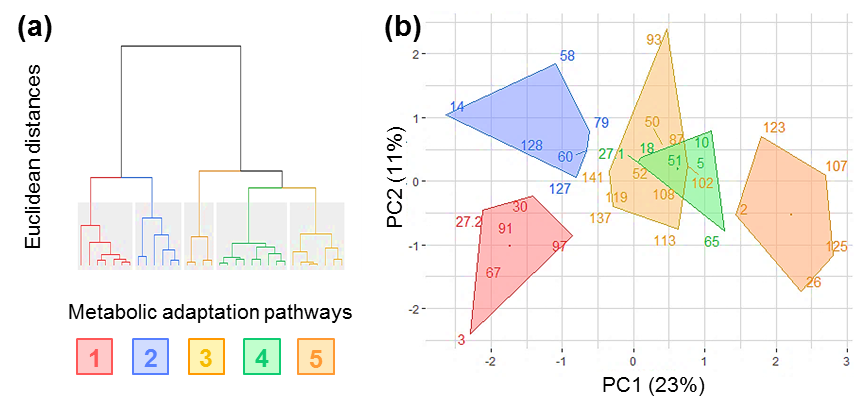

Supplement: Supplementary file 1 [file metabolites-11-00063-s001.zip › SuppFigS1_frenchpaper_Metabolites.png]

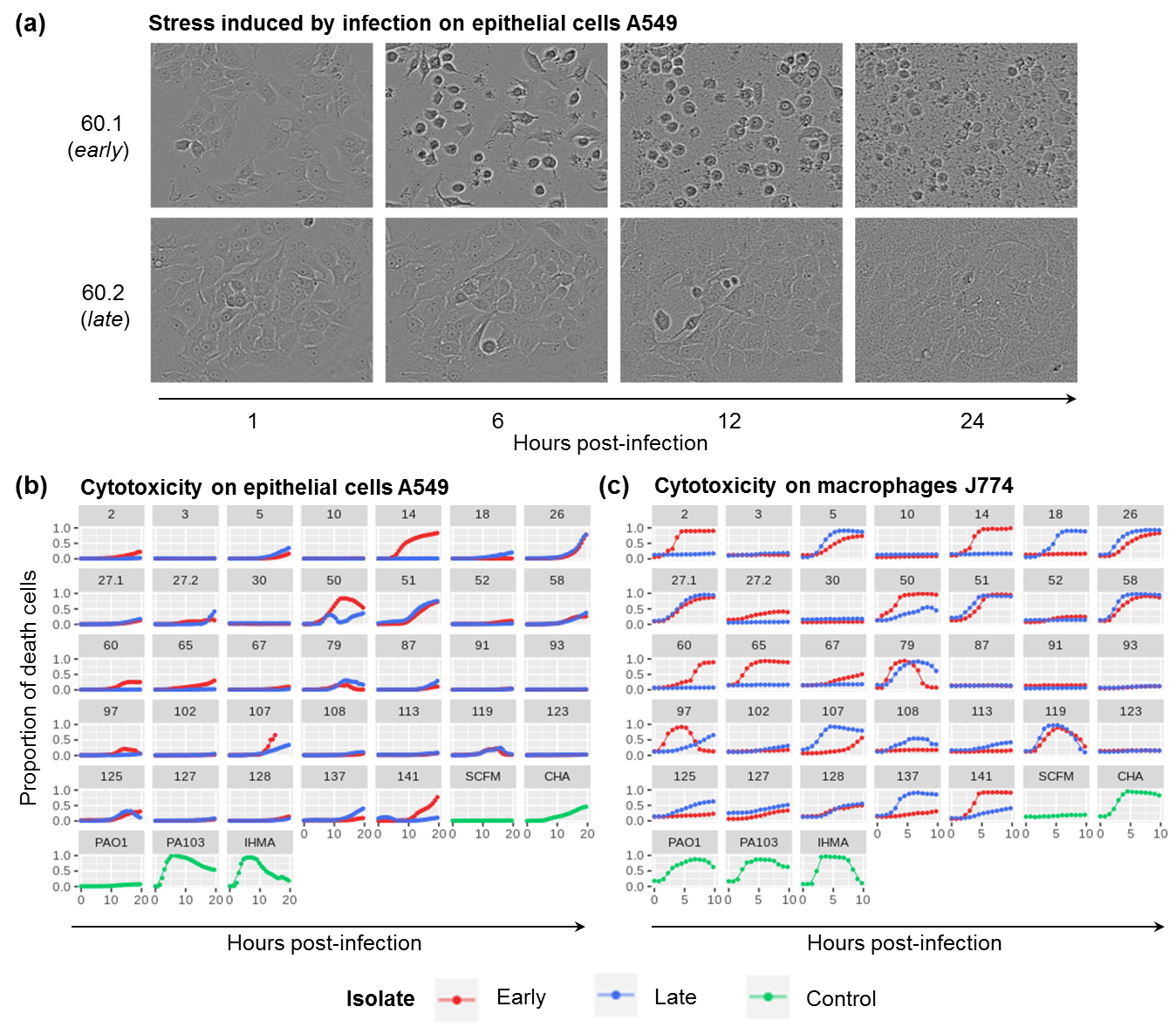

Supplement: Supplementary file 1 [file metabolites-11-00063-s001.zip › SuppFigS2_frenchpaper_Metabolites.png]

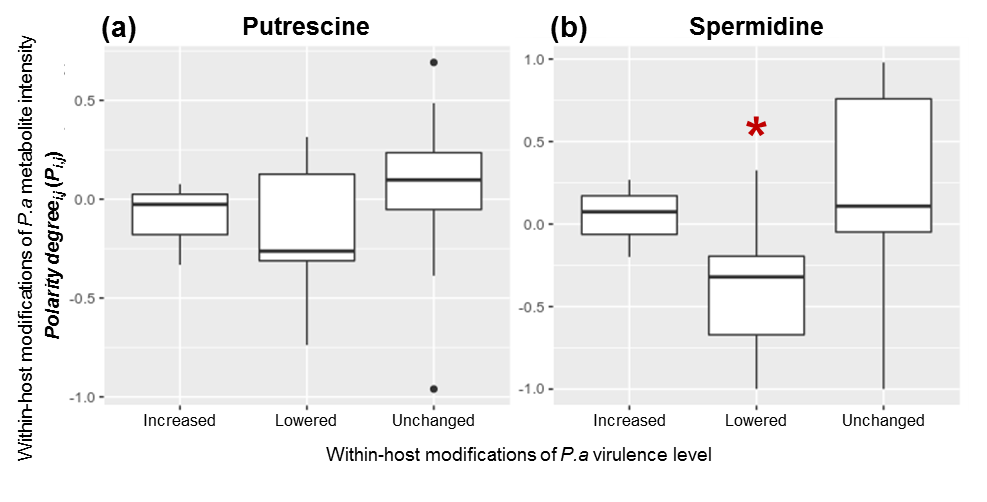

Supplement: Supplementary file 1 [file metabolites-11-00063-s001.zip › SuppFigureS3_Metabolites.png]
